# Delaying arthroplasty: The potential of knee joint distraction in young osteoarthritis patients—A prospective case series

**DOI:** 10.1002/jeo2.70459

**Published:** 2025-10-09

**Authors:** Jasper Vandenrijt, Jozef Michielsen, Jeroen Verhaegen, Pieter Van Dyck, Christiaan H. W. Heusdens

**Affiliations:** ^1^ Faculty of Medicine and Health Sciences University of Antwerp Wilrijk Belgium; ^2^ Department of Orthopedics Antwerp University Hospital Edegem Belgium; ^3^ Orthopaedic Center Antwerp AZ Monica Hospitals Antwerp Belgium; ^4^ Department of Radiology Antwerp University Hospital Edegem Belgium

**Keywords:** cartilage, knee joint distraction, osteoarthritis, orthopaedic surgery

## Abstract

**Purpose:**

Knee joint distraction (KJD) offers a joint preserving alternative for relatively young patients with severe knee osteoarthritis (OA). The aim of this study was to assess the clinical and radiographic success of KJD in patients with severe OA.

**Methods:**

This prospective case series comprised 26 consecutive patients undergoing KJD due to severe knee OA. Clinical and radiographic outcomes were collected at baseline and at 1‐ and 2‐year follow‐up. Complication, reoperation and conversion rates to a primary knee arthroplasty were collected. The patient‐reported outcome measures (PROMs) were the Knee Injury and Osteoarthritis Outcome Score (KOOS), the 36‐item Short Form Health Survey (SF‐36) and the Visual Analogue Scale (VAS) for pain. The radiographs were analysed using an artificial intelligence (AI) based software package to extract compartmental joint space width (JSW), joint space area (JSA) and compartmental imbalance.

**Results:**

Pin tract infections were common, affecting 13 (50%) patients. Five patients (19.2%) received primary knee arthroplasty within the 2‐year follow‐up period. Significant increases (*p* < 0.05) were observed in the JSW and JSA of the most affected compartment, leading to significant improvements in compartmental imbalance. Significant improvements were also observed in all KOOS subscales (*p* < 0.001) which sustained for 2 years. The VAS for pain scores were also significantly improved (*p* < 0.001), as were the SF‐36 subscales (*p* < 0.05) with the exception of the general health subscale.

**Conclusion:**

This case series demonstrates that KJD provides symptomatic relief amongst young patients with knee OA after failed conservative treatment. A significant increase in the JSW and JSA was observed, which may indicate cartilage regeneration. KJD could be an alternative joint‐preserving intervention with potential to delay the need for primary arthroplasty.

**Level of Evidence:**

Level IV, prospective case series.

**Abbreviations**
JSA: joint space area
JSW: joint space width
KJD: knee joint distraction
KOALA: knee osteoarthritis labelling assistant
KOOS: knee injury and osteoarthritis outcome score
OA: osteoarthritis
OARSI‐OMERACT: osteoarthritis research society international standing committee for clinical trials response criteria initiative and the outcome measures in rheumatology
PROMs: patient‐reported outcome measures
SF‐36: short form health survey
TKA: total knee arthroplasty
UKA: unicondylar knee arthroplasty
VAS: visual analogue scale

## INTRODUCTION

Knee osteoarthritis (OA) is a progressive degenerative joint disease characterised by articular cartilage degeneration, subchondral bone remodelling, and soft tissue impairment, causing knee pain, loss of function and disability [6, 16, 26].

Treatment of relatively young patients with knee OA is particularly challenging when non‐surgical therapies fail to provide sufficient symptomatic relief. As there are no other commonly accepted surgical alternatives for patients without a severe malalignment, the majority are being treated with an unicondylar knee arthroplasty (UKA), or total knee arthroplasty (TKA) [9, 17]. Joint registries have shown that 82% of TKAs and 70% of UKAs last for 25 years [6]. Despite high survival rates, one in five patients who receive TKA in the context of knee OA experience chronic post‐surgical pain [3, 5, 24], and the lifetime risk of a revision for patients who receive a primary TKA under the age of 65 is up to 35% [1]. Postponing a TKA by 5 years could potentially avoid 17% TKA revisions [8]. This underscores the need for alternative strategies for relatively young patients with severe knee OA, which ideally alleviates symptoms, promotes cartilage health, and delays or potentially obviates the need for arthroplasty in this population. In this context, KJD is emerging as a joint preserving intervention that may bridge the current treatment gap between non‑surgical treatments and knee arthroplasty.

Clinically, KJD has been associated with significant improvement in knee pain and function [10, 13, 27], thereby postponing the need for primary UKA/TKA with a mean of 5–9 years [15]. A disadvantage of KJD is the occurrence of pin tract infections, reported in 59%–85% of cases [10, 27, 29]. Furthermore, imaging studies have shown increased joint space width (JSW) on X‐ray, and increased cartilage thickness with reduced areas of denuded bone on MRI, indicating that KJD may help to induce cartilage regeneration [12].

Current evidence on KJD remains limited, primarily due to the small number of studies, which often include small sample sizes and short follow‐up periods of less than two years [9]. Additionally, most of the existing research has been conducted at a single research center in the Netherlands [9]. Therefore, more extensive research is warranted. This study aims to evaluate the clinical and radiographic success of KJD by focusing on the joint preservation rate, complications/reoperations, patient‐reported outcome measures (PROMs) and radiographic JSW in a prospective case series with 2 years of follow‐up. The hypothesis was that KJD would result in acceptable complication rates along with improvements in PROMs and JSW.

## MATERIALS AND METHODS

### Patients

This is a prospective single‐center, single‐surgeon, case series at a tertiary referral hospital (Antwerp University Hospital, Belgium). This study was conducted in compliance with the declaration of Helsinki and approved by the institutional review board of Antwerp University Hospital (B300201628769). All study participants signed an informed consent.

Patients with knee OA indicated for primary UKA/TKA after failing a minimum of six months of non‐surgical therapies were considered for KJD if the following inclusion criteria were met: age ≤65 years, symptomatic pain (Visual Analogue Scale (VAS) ≥4) which is explained by the radiographic detectable (Kellgren–Lawrence grade ≥2) knee OA. All patients were indicated for primary UKA/TKA but deemed ineligible owing to their young age (≤65 years). The exclusion criteria were: knee malalignment (>10°) requiring surgical correction, primarily patellofemoral osteoarthritis, inability to wear a distraction frame for six weeks and low molecular weight heparin allergy.

**Figure 1:**
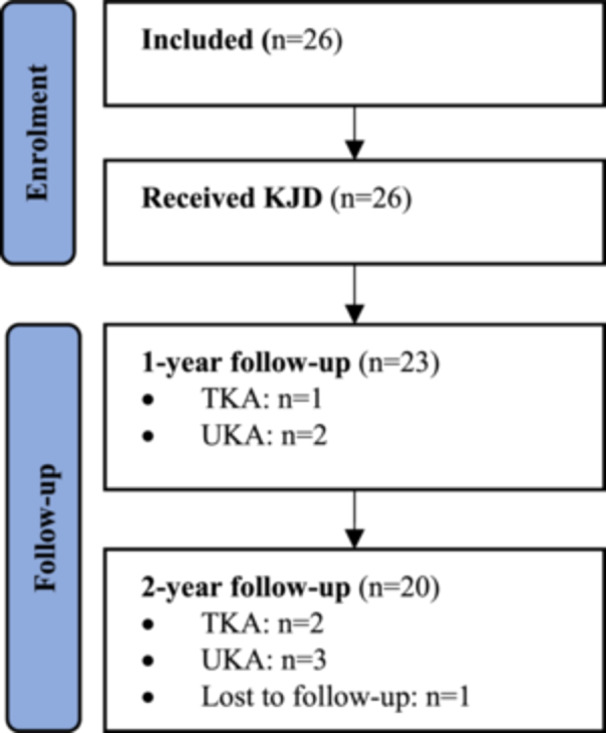
Flow diagram of the included patients included. Five patients (19%) received a TKA/UKA within the 2‐year follow‐up period. One patient (4%) was lost to follow‐up at 2‐year follow‐up. KJD, knee joint distraction; TKA, total knee arthroplasty; UKA, unicondylar knee arthroplasty.

Twenty‐six consecutive patients met the inclusion criteria and were subsequently enroled in the period between May 2016 and May 2021. Baseline characteristics are summarised in Table 1. Notably, three of the included patients (11.5%) had knee OA secondary to previous septic arthritis. A flow chart of included patients is presented in Figure 1, no patients were lost to follow‐up at 2 years.

**Table 1:** Baseline characteristics.

Age (years)	52.2 ± 6.3
Body mass (kg)	92.9 ± 15.1
Height (m)	177.3 ± 8.7
Body mass index (m/kg²)	29.5 ± 4.1
Sex, *n* (%)	
Women	7 (27%)
Man	19 (73%)
Affected knee	
Right	13 (50%)
Left	13 (50%)
Most affected compartment	
Medial	22 (85%)
Lateral	4 (15%)
Indication	
OA	23 (88.5%)
Septic arthritis	3 (11.5%)
Kellgren–Lawrence grade^a^	
0	1 (4%)
1	0 (0%)
2	3 (12%)
3	16 (64%)
4	5 (20%)

*Note*: Values are presented as mean ± SD or number of patients (%).

^a^ One baseline radiograph was taken at a different hospital and was not available for research purposes. The Kellgren–Lawrence grading was performed by the ‘Knee Osteoarthritis Labelling Assistant’ (KOALA, IB Lab GmbH, Vienna, Austria).

### Knee joint distraction

**Figure 2:**
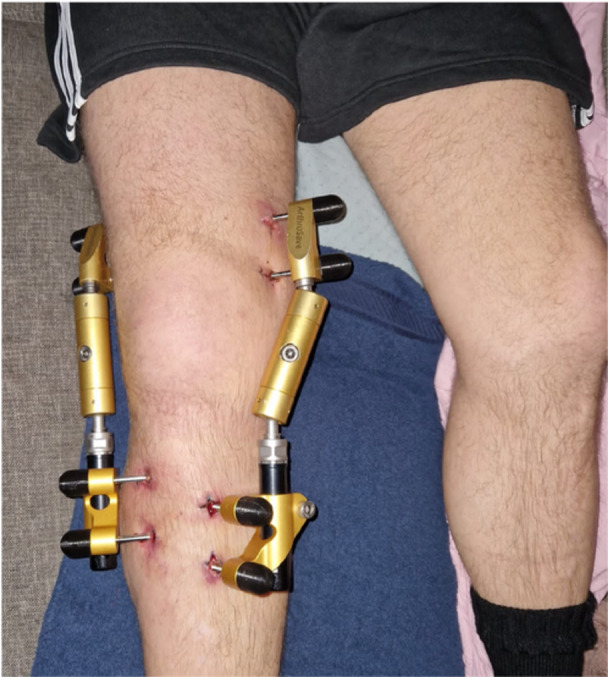
Knee joint distraction (KJD) using the KneeReviver (ArthroSave BV, The Netherlands).

The distraction procedure was performed according to a previously described protocol [10]. Two types of distraction frames were used in this study. The first 13 patients have been treated with the Monotube Triax External Fixation System (Stryker, USA), a device which can perform distraction, but was not originally developed for distraction. During the study the KneeReviver (ArthroSave BV, The Netherlands) was introduced and was used in the remaining patients, as this distraction device was specifically developed for KJD. The frames were positioned both medially and laterally, spanning the knee joint. Each frame was secured with four 5 mm bone pins which were placed in the distal femur and proximal tibia diaphysis bicortical, extra articular, medially and laterally. The knee was manually corrected to a neutral position and the frame was fastened. To prevent neurapraxia, an initial distraction of 3 mm was applied immediately post‐operatively. During a 2 day admission, the distraction was progressively increased with 1 mm increments every 12 h, until 5 mm distraction was achieved (Figure 2). Adequate position of the frames was confirmed radiologically. During the distraction period, weight‐bearing was encouraged, and outpatient follow‐up was organised at 3‐weeks follow‐up. The distraction frames were removed at 6‐weeks follow‐up. This was conducted under general anaesthesia and accompanied by a manipulation of the knee, ensuring no restriction of flexion/extension.

### Radiographic assessment

Standardised weightbearing, anterior‐posterior radiographs were taken at baseline, at 1‑ and 2‑year follow‑up. Knee Osteoarthritis Labelling Assistant (KOALA, IB Lab GmbH, Vienna, Austria), an automated artificial intelligence based software package, was used to analyse the images.

**Figure 3:**
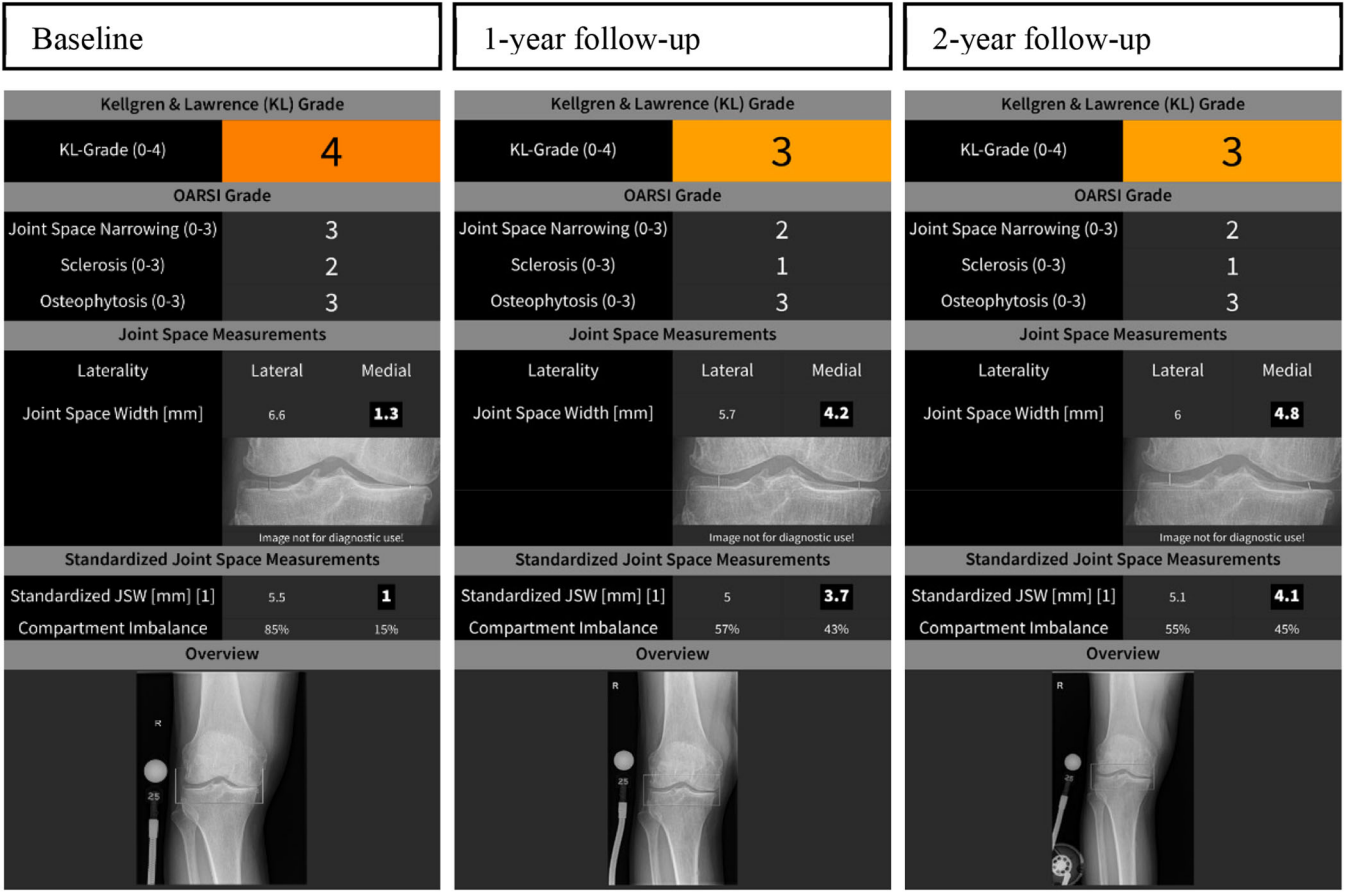
Illustration of the reports generated by the ‘Knee Osteoarthritis Labelling Assistant’ (KOALA, IB Lab GmbH, Vienna, Austria). These reports present the evolution of a single knee joint distraction (KJD) patient's radiographs from baseline (pre‐KJD) to 1‐ and 2‐year post‐KJD.

Using KOALA, measures of minimal and standardised JSW as well as joint space area (JSA) were obtained for the most‐affected compartment and least‐affected compartment. The standardised JSW is derived by normalising the JSW according to the width of the tibia. This normalisation enhances the sensitivity of JSW, by mitigating the variance caused by differences in the height of patients [23]. Subsequently, the ratio between the standardised medial and lateral JSW was used to ascertain compartmental imbalance. According to a previous study, an anatomical medial/lateral balance of 45/55% to 50/50% can be considered ideal [14]. Furthermore, KOALA also provided the Osteoarthritis Research Society International (OARSI) grades for sclerosis, osteophytes, and joint space narrowing, which served as inputs to compute a whole‐joint Kellgren–Lawrence grade. Example radiographic reports, produced by KOALA, are shown in Figure 3.

### Clinical assessment

Adverse events and additional procedures were prospectively recorded and graded according to the Clavien–Dindo classification system for surgical complications [5]. Minor complications include Grade I and II, which comprise complications requiring no intervention beyond the standard of care (Grade I) and complications that need pharmacological treatment (Grade II). Major complications include Grade III, IV and V, which involves complications that require surgical procedures (Grade IIIa + IIIb), intensive care management (Grade IV), and result in death (Grade V) [5].

Clinical outcome was assessed through self‐reported questionnaires, completed on paper by the study patients at baseline, 1‐year and 2‐year follow‐up. Questionnaire endpoints included the Knee Injury and Osteoarthritis Outcome Score (KOOS), a 100 mm Visual Analogue Scale (VAS) for pain in rest and during exercise and the 36‐item Short Form Health Survey (SF‐36). The SF‐36 comprises three concepts of health‐related quality of life (physical status, mental well‐being and general health) and is divided into eight subscales: physical functioning, role limitations due to physical health, role limitations due to emotional problems, vitality, emotional well‐being, social functioning, pain and general health [30].

### Statistical analysis

All statistical analyses were performed in JMP® Pro Version 17 (SAS Institute Inc., Cary, North Carolina, USA). Patient characteristics were summarised using descriptive statistics and presented as mean ± SD or as *n* (%) for discrete variables. A linear mixed model with time as a fixed effect and patient as random intercept was used to assess clinical and radiographic outcomes over time. In case of a significant time effect, a post hoc comparison was conducted between the baseline and post‑operative timepoints using a Dunnett's test for multiple comparisons. Baseline outcomes were compared between patients who underwent TKA/UKA within the 2‐year follow‐up and those who did not, and a comparison was made between patients with and without pin tract infections at 1‐ and 2‐year follow‐up using a Mann–Whitney *U* test due to the small patient numbers. To evaluate treatment (non‐)response, the Osteoarthritis Research Society International (OARSI) Standing Committee for Clinical Trials Response Criteria Initiative and the Outcome Measures in Rheumatology (OMERACT) responder criteria were applied on the KOOS scores. A positive response is defined by (i) an improvement in KOOS Pain or KOOS ADL of ≥50% with an absolute change of ≥20, or (ii) an improvement of ≥20% with an absolute change of ≥10 in both KOOS pain and KOOS ADL [25]. Patients who received knee replacement surgery during follow‐up were automatically classified as non‑responders. For all statistical tests a *p* < 0.05 was deemed statistically significant.

A power analysis was conducted to determine the required sample size to detect a minimal clinically important difference of 18 points in KOOS pain [19], with a standard deviation of 21 points [28] using a paired‐sample t‐test. Assuming a significance level of 5% and statistical power of 80%, a minimal sample size of 13 patients would be needed.

## RESULTS

### Complications/additional procedures

Thirteen patients (50%) sustained a single or multiple pin tract infections during the distraction period, which were resolved with oral antibiotics (Grade II). In one patient (3.8%), scar tissue adhered to the tibia at a pin wound site after bone pin removal. A scar tissue release was performed under local anaesthesia (Grade IIIa). Two patients (7.7%) experienced stiffness due to arthrofibrosis requiring mobilisation under anaesthesia (Grade IIIb), improving limited range of motion to full range of motion at final follow‐up.

Eight patients (30.8%), received an intra‐articular injection (7 hyaluronic acid; 1 CM‐Chitosan) within the 2‐years of follow‐up. Of those eight, five patients (19.2%) ultimately required conversion to UKA (*n* = 3) or TKA (*n* = 2) within 2‐years follow‐up, due to insufficient symptomatic relief.

### Radiographic outcomes

**Figure 4:**
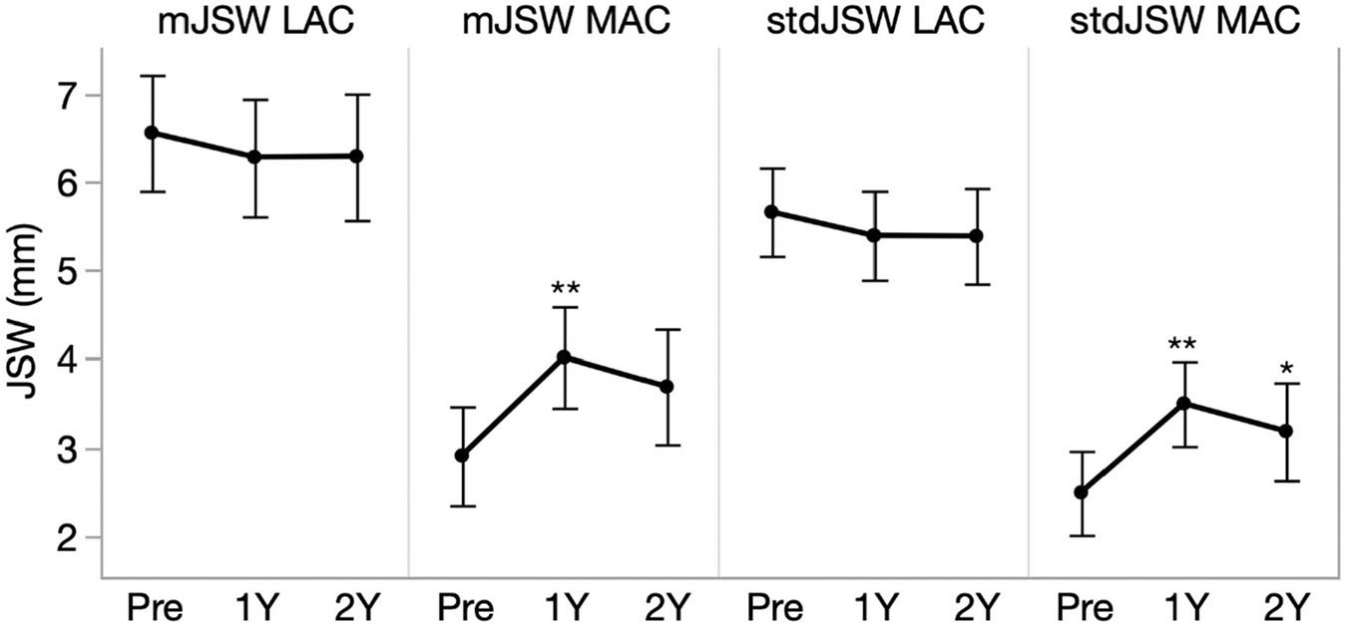
The least square (LS) means estimates for the minimal (mJSW) and standardised joint space width (stdJSW) of the most (MAC) and least affected compartment (LAC) at baseline, 1‐year and 2‐year follow‐up. The error bars are constructed using 95% confidence interval [CI]. *p < 0.05; **p < 0.01.

Structural changes were observed in the most affected compartment over time (Table 2). The minimal JSW increased by 38.6% (1.12 mm, 95% CI [[0.41–1.82]) at 1‐year follow‐up (*p* = 0.0014), and the standardised JSW also increased by 41.1% (1.01 mm, 95% CI [0.40–1.63]) at 1‐year follow‐up (*p* = 0.0009), and 28.2% (0.70 mm, 95% CI [0.003–1.39]) at 2‐year follow‐up (*p* = 0.0487) (Figure 4).

**Table 2:** Comparison between radiological outcomes at baseline and follow‐up.

				Fixed effect (time)	Post hoc comparison			
					Baseline − 1Y		Baseline − 2Y	
	Baseline	1Y	2Y	*p* value	Mean difference [95% CI]	Adjusted *p*‐value	Mean difference [95% CI]	Adjusted *p* value
mJSW MAC	2.90 ± 0.28	4.02 ± 0.29	3.69 ± 0.33	**0.0024**	1.12 [0.41–1.82]	**0.0014**	0.79 [−0.01, 1.58]	0.0539
mJSW LAC	6.56 ± 0.33	6.28 ± 0.33	629 ± 0.36	0.4793				
stdJSW MAC	2.48 ± 0.24	3.50 ± 0.24	3.18 ± 0.28	**0.0016**	1.01 [0.40–1.63]	**0.0009**	0.70 [0.003–1.39]	**0.0487**
stdJSW LAC	5.66 ± 0.25	5.40 ± 0.25	5.39 ± 0.27	0.2791				
JSA MAC	101.10 ± 5.56	119.62 ± 5.66	115.18 ± 6.55	**0.0156**	18.52 [4.00–33.04]	**0.0106**	14.08 [−2.37, 30.52]	0.1022
JSA LAC	164.38 ± 8.88	166.82 ± 8.93	167.29 ± 9.49	0.8805				
IMB MAC	29.29 ± 2.05	39.32 ± 2.09	36.35 ± 2.39	**0.0002**	10.03 [4.98–15.08]	**<0.0001**	7.06 [1.33–12.79]	**0.0138**
IMB LAC	70.71 ± 2.05	60.68 ± 2.09	63.65 ± 2.39	**0.0002**	−10.03 [−4.98, −15.08]	**<0.0001**	−7.06 [−1.33, −12.79]	**0.0138**

*Note*: Baseline, 1Y and 2Y scores are presented as mean ± SE and are based on the least squares means estimates. The post hoc comparisons are conducted using a Dunnett's for multiple comparison and *p*‐values, mean differences and 95% CIs are presented. Bold values indicate statistically significant effects.

Abbreviations: IMB, compartmental imbalance; JSA, joint space area; LAC, least affected compartment; MAC, most affected compartment; mJSW, minimal joint space width; stdJSW, standardised joint space width.

**Figure 5:**
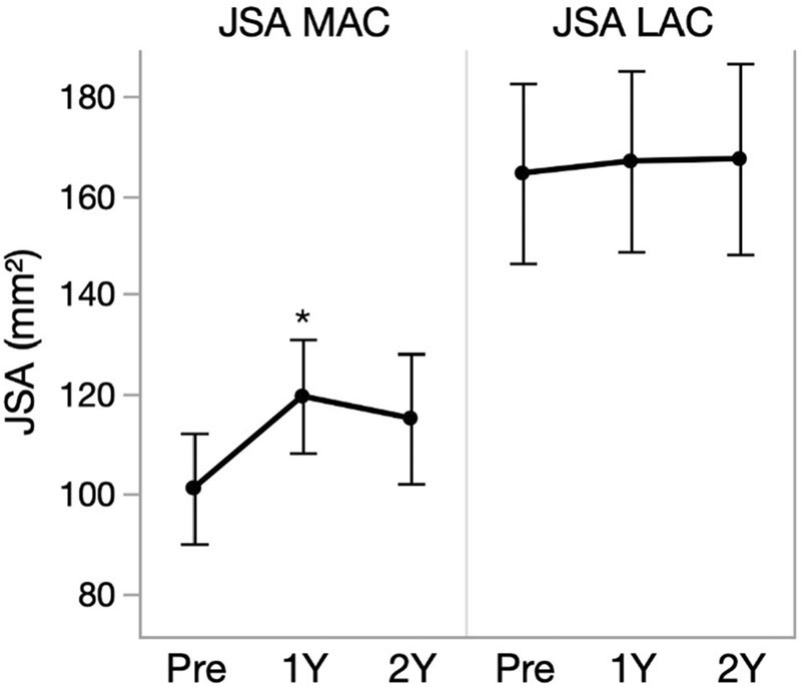
The least square (LS) means estimates for the joint space area (JSA) of the most (MAC) and least affected compartment (LAC) at baseline, 1‐year and 2‐year follow‐up. The error bars are constructed using 95% CI. *p < 0.05; **p < 0.01.

JSA increased by 18.3% (18.52 mm², 95% CI [4.00–33.04]) at 1‐year follow‐up (*p* = 0.0106) (Figure 5). Regarding the compartmental balance, there was a shift towards a more optimal balance (most affected compartment/least affected compartment %) from 29.27/70.73% to 39.32/60.68% (*p* < 0.0001) and 36.35/63.65% (*p* = 0.0138) at 1‐ and 2‐year follow‐up respectively.

The Kellgren–Lawrence grade improved in 39.1% of patients (*n* = 9/23) at 1‐year follow‐up, and in 31.3% of patients at 2‐year follow‐up (*n* = 5/17).

### PROMs

An overview of the PROMs is presented in Table 3. Significant time effects (*p* < 0.05 for all outcomes) were detected by the linear mixed models for all KOOS subscales. Subsequent post‐hoc testing revealed significant improvements at both time points compared to baseline for all KOOS subscales.

**Table 3:** Comparison between patient‐reported outcomes at baseline with follow‐up.

				Fixed effect	Post hoc comparison			
				(Time)	Baseline − 1Y		Baseline − 2Y	
	Baseline	1Y	2Y	*p* value	Mean difference [95% CI]	Adjusted *p*‐value	Mean difference [95% CI]	Adjusted *p* value
KOOS								
Pain	40.6 ± 3.7	69.6 ± 3.9	68.8 ± 4.3	**<0.0001**	29.0 [19.5–38.5]	**<0.0001**	28.2 [17.8–38.7]	**<0.0001**
Sym	48.2 ± 3.6	68.0 ± 3.8	67.1 ± 4.1	**<0.0001**	19.7 [11.1–28.4]	**<0.0001**	18.8 [9.5–28.2]	**<0.0001**
ADL	45.2 ± 3.8	71.7 ± 4.0	74.3 ± 4.4	**<0.0001**	26.5 [16.6–36.5]	**<0.0001**	29.1 [18.4–39.8]	**<0.0001**
Sports/Rec	12.6 ± 4.1	34.8 ± 4.4	33.2 ± 4.7	**<0.0001**	22.2 [11.7–32.7]	**<0.0001**	20.5 [9.2–31.8]	**0.0003**
QoL	25.6 ± 3.4	44.5 ± 3.6	44.8 ± 3.9	**<0.0001**	18.9 [9.6–28.1]	**<0.0001**	19.2 [9.2–29.1]	**0.0001**
SF‐36								
PF	35.8 ± 4.0	58.8 ± 4.2	59.3 ± 4.5	**<0.0001**	23.0 [13.7–32.4]	**<0.0001**	23.5 [13.5–33.6]	**<0.0001**
RP	25.9 ± 8.0	64.7 ± 8.5	66.6 ± 9.2	**<0.0001**	38.8 [18.4–59.1]	**0.0002**	40.7 [18.8–62.7]	**0.0002**
RE	60.5 ± 7.7	82.1 ± 8.1	79.1 ± 8.8	**0.0393**	21.6 [1.5–41.7]	**0.0337**	18.6 [−3.1, 40.3]	NS
VT	56.8 ± 4.1	71.0 ± 4.3	68.7 ± 4.5	**0.0011**	14.3 [5.7–22.8]	**0.0009**	11.9 [2.7–21.2]	**0.0099**
MH	70.8 ± 3.2	82.1 ± 3.3	78.9 ± 3.4	**0.0002**	11.3 [5.6–17.0]	**0.0001**	8.1 [1.9–14.3]	**0.0088**
SF	66.1 ± 5.3	77.8 ± 5.5	82.0 ± 5.9	**0.0113**	11.7 [0.3–23.0]	**0.0439**	15.9 [3.6–28.2]	**0.0098**
BP	41.8 ± 4.5	64.5 ± 4.8	68.5 ± 5.4	**0.0002**	22.7 [8.8–36.6]	**0.0011**	26.7 [11.8–41.6]	**0.0004**
GH	60.4 ± 4.0	71.3 ± 4.3	67.2 ± 4.7	NS				
HC	39.9 ± 4.8	73.8 ± 5.1	59.3 ± 5.7	**0.0002**	33.9 [16.8–51.1]	**<0.0001**	19.4 [1.3–37.4]	**0.0335**
VAS								
Rest	28.8 ± 4.4	11.1 ± 4.2	6.9 ± 4.8	**0.0005**	−17.7 [−29.2, −6.3]	**0.0022**	−21.9 [−34.4, −9.4]	**0.0007**
Exercise	73.0 ± 4.6	40.9 ± 4.8	32.8 ± 5.5	**<0.0001**	−32.1 [−45.8, −18.4]	**<0.0001**	−40.2 [−55.3, −25.1]	**<0.0001**

*Note*: Baseline, 1Y and 2Y scores are presented as mean ± SE and are based on the least squares means estimates. The post hoc comparisons are conducted using a Dunnett's for multiple comparison and *p* values, mean differences and 95% CIs are presented. Bold values indicate statistically significant effects.

Abbreviations: ADL, activities daily living; BP, bodily pain; CIs, confidence intervals; GH, general health perception; HC, health change; KOOS, Knee Injury and Osteoarthritis Outcome Score; MH, mental health; PF, physical functioning; QoL, Quality of Life; RE, role‐emotional; RP, role‐physical; SF, social functioning; SF‐36, 36‐Item Short Form Survey; Sport/Rec, sports and recreation; Sym, symptoms; VAS, Visual Analogue Scale; VT, vitality.

A similar linear mixed model was fitted for the VAS for pain and SF‐36. Significant time effects (*p* < 0.05 for al outcomes) were evident in all models, except for the global health subscale of the SF‐36. The post hoc tests revealed significant improvements at both 1‐ and 2‐year follow‐up compared with baseline across all scores, except for the Role‐Emotional subscale of the SF‐36 questionnaire which only showed a significant improvement at 1‐year follow‐up.

On an individual level, 62.5% could be considered a clinical responder at 1‐year follow‐up, and 63.6% at 2‐year follow‐up, as defined by the OARSI‐OMERACT criteria. Of the eight patients (30.8%) who received an intra‐articular injection during follow‐up, none met the clinical responder criteria before the injection, and seven failed to achieve the responder criteria after the injection.

### Subgroup analysis

Comparing baseline PROMs between the patients who underwent primary knee arthroplasty during follow‐up with those who did not, revealed worse baseline scores in the role limitations due to emotional problems and social functioning subscales of the SF‐36 (Table 4).

**Table 4:** Subgroup analysis.

	No UKA/TKA (*n* = 20)	UKA/TKA (*n* = 5)	*p* value
KOOS			
Pain	43.30 ± 12.59	27.20 ± 16.89	0.066
Sym	49.65 ± 17.17	39.20 ± 8.93	0.124
ADL	48.00 ± 16.13	31.20 ± 19.60	0.102
Sports/Rec	14.30 ± 15.06	3.40 ± 7.60	0.065
QoL	28.40 ± 15.69	12.60 ± 9.02	0.080
SF‐36			
PF	37.75 ± 17.58	24.00 ± 12.45	0.107
RP	32.50 ± 42.22	0.00 ± 0.00	0.053
RE	70.00 ± 43.13	20.00 ± 44.72	**0.032**
VT	57.75 ± 22.51	50.00 ± 29.79	0.655
MH	73.60 ± 16.84	58.40 ± 21.28	0.133
SF	73.13 ± 21.94	35.00 ± 27.10	**0.012**
BP	45.33 ± 23.05	27.00 ± 29.50	0.165
GH	61.75 ± 21.04	53.00 ± 32.90	0.562
HC	42.50 ± 25.78	30.00 ± 20.92	0.423
VAS			
Rest	2.33 ± 2.35	5.67 ± 2.52	0.057
Exercise	7.12 ± 1.36	8.00 ± 1.63	0.323

*Note*: Comparison of the baseline scores, presented as mean ± SD, between patients who progressed to primary knee arthroplasty and patients who did not. Bold values indicate statistically significant effects.

Abbreviations: ADL, activities daily living; BP, bodily pain; CIs, confidence intervals; GH, general health perception; HC, health change; KOOS, Knee Injury and Osteoarthritis Outcome Score; MH, mental health; PF, physical functioning; RE, role‐emotional; RP, role‐physical; SD, standard deviation; SF, social functioning; SF‐36, 36‐Item Short Form Survey; Sport/Rec, sports and recreation; Sym, symptoms; TKA, total knee arthroplasty; UKA, unicondylar knee arthroplasty; VAS, Visual Analogue Scale; VT, vitality.

During follow‐up, no significant differences were observed in the KOOS scores between patients who did and did not suffer from pin tract infection at 1‐ and 2‐year follow‐up.

## DISCUSSION

This prospective case series investigated the clinical and radiographic outcomes of KJD in relatively young patients with knee OA, who were unresponsive to conservative therapies. Both clinical and structural improvements were observed at 1‑year follow‐up, which were sustained through the 2‐year follow‐up period. The PROMs improved significantly, and on an individual basis, 62.5% and 63.6% of patients were classified as clinical responders at 1‐ and 2‐year follow‐up, respectively, demonstrating the clinical relevance of the findings. Frequent complications were superficial pin tract infections, affecting 50% of the patients. Five patients (19%) experienced insufficient symptomatic relief post‐KJD and required a primary knee arthroplasty within the 2‐year follow‐up period.

There is a growing need for alternative treatment strategies for relatively young patients with severe knee OA. KJD has been introduced as an alternative joint‐preserving intervention. Most of the current research on KJD has been conducted by a single research center in the Netherlands, including two randomised controlled trials (RCTs) comparing KJD with TKA and high tibial osteotomy [9]. These studies showed that KJD was non‐inferior to TKA and high tibial osteotomy in terms of clinical improvement [28, 29]. However, HTO is generally indicated for patients with significant malalignment and mild‐to‐moderate OA (Kellgren–Lawrence grade 2–3), and is less suitable for end‐stage (Grade 4) OA [18]. In contrast, KJD is suitable for patients with end‐stage (Grade 4) OA, but not recommended in cases of pronounced malalignment (greater than 10°) [29]. Therefore, HTO and KJD may serve complementary roles: while HTO is suited for malaligned knees, KJD may bridge the gap between non‐surgical interventions and UKA/TKA in a young OA patients with well‐aligned knees.

In this study, automated AI‐based software was used to analyse the knee radiographs, enhancing inter‐observer agreement [21, 22]. This study, demonstrated improvements in JSW and JSA, suggesting potential cartilage regeneration. However, a precise assessment of post‐KJD cartilage health demands morphological high‐resolution 3D cartilage specific sequences, such as the spoiled gradient recalled echo and dual echo steady‐state sequences. Using dGEMRIC in a cohort of 20 patients, Besselink et al. found no significant change in cartilaginous tissue between baseline and two years following KJD [2]. It is important to note that dGEMRIC may be inaccurate in late‐stage OA, and better suited to assess early OA changes [20]. In contrast, Jansen et al. reported improvements in MRI based cartilage thickness and denuded bone between baseline and 2‐years follow‐up, using 1.5 T and 3 T MRIs with a 3D spoiled gradient recalled echo with fat suppression [11].

Pin tract infections were common (50%), but successfully managed with oral antibiotic therapy. This allowed treatment completion without impacting post‐operative PROMs. Pin tract infections have not been associated with an increased risk of peri‐prosthetic joint infection in patients converting to knee arthroplasty. Major complications requiring a surgical intervention were less frequent. One patient (3.8%) required scar‐tissue release under local anaesthesia, and two patients (7.7%) underwent mobilisation under anaesthesia due to arthrofibrosis. Importantly, the joint preservation rate in our cohort was 81%, with 19% of patients ultimately requiring conversion to TKA/UKA. Interestingly, patients who received primary knee arthroplasty in the first two years following KJD exhibited significantly worse baseline scores on the SF‐36 subscales related to mental well‐being (social functioning and role limitations due to emotional problems).

Previous studies consistently excluded patients with a history of septic arthritis. Our cohort included three patients (11.5%) with knee OA secondary to previous septic arthritis. For these patients, KJD resulted in good clinical results, and, apart from pin tract infections in two patients, was uncomplicated.

This study has limitations. First, the sample size is relatively small, complicating subgroup analysis to identify effect modifiers. Second, as this study is a case series, no direct comparison can be made between KJD and alternative treatments. Third, patients were recruited at a single research center, which could bias the representativeness of our study sample. Most of the existing research on KJD has been conducted by a single research center in the Netherlands. A notable strength of this study is that it is the first to be conducted by a different research center. Fourth, throughout the study a transition from the Monotube Triax External Fixation System (Stryker, Switzerland) to the purpose‐built KneeReviver (ArthroSave BV, The Netherlands) was made. However, the sample size was too small to assess potential differences between both distraction frames. Finally, this study is limited by the 2‐year follow‐up results of KJD. To investigate whether KJD can successfully postpone a primary knee arthroplasty long‐term follow‐up studies are required.

## CONCLUSION

To conclude, this case series demonstrated that KJD provides symptomatic relief amongst young patients with knee OA after failed conservative treatment. A significant increase in the JSW and JSA was observed, which may indicate cartilage regeneration. KJD could be an alternative joint preserving intervention, with potential to postpone primary knee arthroplasty for young patients with knee OA.

## AUTHOR CONTRIBUTIONS

All authors contributed to conceptualisation and design. Data collection was performed by Christiaan H. W. Heusdens and Jasper Vandenrijt. Data analysis was performed by Jasper Vandenrijt. The first draft of the manuscript was written by Jasper Vandenrijt and Christiaan H. W. Heusdens. Previous versions of the manuscript were thoroughly revised by all authors and the final manuscript was also read and approved by all authors.

## CONFLICT OF INTEREST STATEMENT

The authors declare no conflicts of interest.

## ETHICS STATEMENT

All procedures performed in studies involving human participants were in accordance with the ethical standards of the institutional research committee of the Antwerp University Hospital (B300201628769, 16/18/207) and with the 1964 Helsinki declaration and its later amendments or comparable ethical standards. Informed consent was obtained from all individual participants included in the study.

## Data Availability

The collected and analysed data collected are not publicly available.
